# A Two-Step Method for Dynamic Parameter Identification of Indy7 Collaborative Robot Manipulator

**DOI:** 10.3390/s22249708

**Published:** 2022-12-11

**Authors:** Meseret Tadese, Nabih Pico, Sungwon Seo, Hyungpil Moon

**Affiliations:** 1 Department of Mechanical Engineering, Sungkyunkwan University, Suwon 16419, Gyeonggi-do, Republic of Korea; 2 Facultad de Ingeniería en Electricidad y Computación, Escuela Superior Politécnica del Litoral, ESPOL, Campus Gustavo Galindo, Guayaquil 09-01-5863, Ecuador

**Keywords:** dynamic parameters identification, friction identification, friction model, robot dynamics

## Abstract

Accurate dynamic model is critical for collaborative robots to achieve satisfactory performance in model-based control or other applications such as dynamic simulation and external torque estimation. Such dynamic models are frequently restricted to identifying important system parameters and compensating for nonlinear terms. Friction, as a primary nonlinear element in robotics, has a significant impact on model accuracy. In this paper, a reliable dynamic friction model, which incorporates the influence of temperature fluctuation on the robot joint friction, is utilized to increase the accuracy of identified dynamic parameters. First, robot joint friction is investigated. Extensive test series are performed in the full velocity operating range at temperatures ranging from 19 °C to 51 °C to investigate friction dependency on joint module temperature. Then, dynamic parameter identification is performed using an inverse dynamics identification model and weighted least squares regression constrained to the feasible space, guaranteeing the optimal solution. Using the identified friction model parameters, the friction torque is computed for measured robot joint velocity and temperature. Friction torque is subtracted from the measured torque, and a non-friction torque is used to identify dynamic parameters. Finally, the proposed notion is validated experimentally on the Indy7 collaborative robot manipulator, and the results show that the dynamic model with parameters identified using the proposed method outperforms the dynamic model with parameters identified using the conventional method in tracking measured torque, with a relative improvement of up to 70.37%.

## 1. Introduction

The control of robotic systems mainly depends on accurate dynamic models to increase performance. The topic has been covered in recent studies as it relates to control approaches (such as sliding mode control, model predictive control, passivity-based control, and state-space control) for a variety of systems [1,2,3,4,5] having common explicit dynamic models in the control loop. The accuracy of the dynamic parameters that characterize the dynamic model is the most important aspect affecting the dynamic model’s accuracy. Such dynamic parameters are typically unknown or only partially known via nominal parameters approximately calculated from computer-aided design (CAD) software. Because of manufacturing limitations, such parameters’ accuracy cannot be guaranteed [6]. An identification method is an alternate approach for estimating dynamic parameters (such as mass, the center of mass, and moment of inertia) employing special experiments. This method has been used and researched extensively in [6,7,8].

The most prevalent strategy for experimental identification methods is a combined Inverse Dynamics Identification Model and Least Squares (IDIM-LS) method. The accuracy of parameter estimations for such an approach is often affected by measurement noise and modeling errors. Significant research efforts have been made on identifying appropriate exciting trajectories to overcome measurement noise concerns [9,10,11]. However, the majority of modeling errors that results in considerable deterministic structural errors are caused by disregarding complex and nonlinear joint dynamics effects (such as friction), which cannot be explained by random variables. For example, strain-wave transmission methods like the Harmonic Drive (HD), which are frequently used in collaborative robot manipulators because of their benefits of high-quality characteristics of loading capability and lightweight, can cause nonlinear joint dynamics.

Friction is one of the most prominent undesired nonlinear phenomena inherent in HD, introducing modeling errors and affecting the accuracy of the dynamic model. Although assuming the friction model as Coulomb friction plus viscous friction linear to the robot joint velocity [12] is a common method for dynamic model identification, it is generally insufficient in practice. Furthermore, comparing two tests, one at robot startup and one after the thermal transient owing to friction change with robot joint temperature variation, will result in a model discrepancy. In general, since friction is influenced by temperature [13,14,15,16,17], improving the accuracy of the friction force model will significantly improve the accuracy of the robot dynamic model.

The present research aims to realize the dynamic parameter identification of a manipulator as accurately as possible. The primary contribution is using a reliable dynamic friction model to increase the accuracy of the identified dynamic parameters. Considering the influence of robot joint temperature variation on robot joint friction, the proposed method aims to increase the accuracy of dynamic parameters by minimizing non-structural uncertainty through the use of a more accurate dynamic friction model. The important contributions that this work has made can be summed up as follows:

- a linear parametrization that describes the dynamics of the Indy7 collaborative robot manipulator has been developed,
- the minimum set of base parameters needed to characterize the dynamic behavior of the Indy7 robot has been successfully approximated,
- during robot dynamic parameter identifications, a collaborative robot’s inherently nonlinear joint dynamics were taken into account,
- validation of the accuracy of the torque predictions made by the identified robot dynamic model.

The remainder of the paper is organized as follows: Section 2 presents the mathematical model of the robot manipulator and the development of a linear identification model and friction model. Section 3 discusses the identification experiment process and compares the accuracy between two dynamic models with parameters identified using conventional and proposed linear identification models. After analyzing validation results, the research outcome is discussed and summarized in Section 4 and Section 5, respectively.

## 2. Dynamic Modeling

The inverse dynamic model of a rigid robot composed of *N* moving links calculates the motor torque vector $\tau_{m}$ (the control input) as a function of the generalized coordinates. It can be obtained from the Lagrangian or Newton Euler equation as recalled in [18,19]:

(1) $$M\left(q\right)+C\left(q,\dot{q}\right)\dot{q}+G\left(q\right)=\tau_{m}$$

where $q\in R^{N}$ represents the *N* joint angles, M(q) is the inertia matrix, $C(q,\dot{q})$ captures the centrifugal and Coriolis effects, and G(q) represents the gravitational torque. The drive torque $\tau_{m}$ applied by the joint motor controls the system.

### 2.1. Linear Identification Model

Using the barycentric parameters [20] or the modified Newton-Euler parameters [21] a model of the type in Equation (1) may also be parameterized and transformed into a model that is linear in a new set of unknown dynamic parameters $\Phi_{s}$.

(2) $$\tau_{m}=Y_{s}\left(q,\dot{q},\ddot{q}\right)\Phi_{s}$$

where $Y_{s}\left(q,\dot{q},\ddot{q}\right)\in R^{N\times 11N}$ is the observation matrix, which depends only on the motion data, and $\Phi_{s}\in R^{11N\times 1}$ is a vector of standard dynamic parameters:

$$\begin{array}{l} \Phi_{s}={\left[\Phi_{1}^{T}\cdots \Phi_{N}^{T}\right]}^{T},\\ \Phi_{i}={\left[Ixx_{i},Ixy_{i},Ixz_{i},Iyy_{i},Iyz_{i},Izz_{i},mx_{i},my_{i},mz_{i},m_{i},Ia_{i}\right]}^{T} \end{array}$$

For each link *i*, it comprised the six components of the inertia tensor, ($Ixx_{i},Ixy_{i},Ixz_{i},Iyy_{i},$$Iyz_{i},Izz_{i}$); the three components of the first moment, ($mx_{i},my_{i},mz_{i}$); the mass, $m_{i}$; and the total inertia moment for rotor actuator and gears, $Ia_{i}$.

Because friction is the primary element influencing dynamic model accuracy, a comprehensive dynamic model must contain friction in addition to the fundamental dynamic mathematical model Equation (1). In the conventional dynamic parameter identification method [12], dynamic parameters have been expanded as shown in Equation (4) to include the most familiar static friction model Equation (3).

(3) $$\begin{array}{cc} \; & \tau_{s}=F_{c}\mathrm{sign}(\dot{q})+F_{v}\dot{q}+F_{o} \end{array}$$

(4) $$\begin{array}{cc} \; & {\bar{\Phi }}_{i}={\left[\Phi_{i}^{T},F_{c,i},F_{v,i},F_{o,i}\right]}^{T}\\ \; & {\bar{\Phi }}_{s}={\left[{\bar{\Phi }}_{1}^{T}\cdots {\bar{\Phi }}_{N}^{T}\right]}^{T},\;\in R^{14N\times 1}\\ \; & {\bar{Y}}_{s}\left(q,\dot{q},\ddot{q}\right)=\left[Y_{s}\left(q,\dot{q},\ddot{q}\right)\;diag\left(\mathrm{sign}(\dot{q})\right)\;diag\left(\dot{q}\right)\;I_{N\times N}\right] \end{array}$$

(5) $$\begin{array}{cc} \; & \tau_{m}={\bar{Y}}_{s}\left(q,\dot{q},\ddot{q}\right){\bar{\Phi }}_{s} \end{array}$$

where $\tau_{s}$ is a friction torque, $F_{c}=diag\left\{F_{c,1}\;\cdots \;F_{c,N}\right\}$ is the Coulomb friction, $F_{v}=diag\left\{F_{v,1}\;\cdots \;F_{v,N}\right\}$ is the viscous friction, and $F_{o}=diag\left\{F_{o,1}\;\cdots \;F_{o,N}\right\}$ is the offset friction torque. $\mathrm{sign}(\dot{q})={\left[sign\left({\dot{q}}_{1}\right)\;\cdots \;sign\left({\dot{q}}_{N}\right)\right]}^{T}$ is a signum function defined such that $sign({\dot{q}}_{i})=1$ if ${\dot{q}}_{i}>0,\;sign\left({\dot{q}}_{i}\right)=0$ if ${\dot{q}}_{i}=0,$ and $sign({\dot{q}}_{i})=-1$ if ${\dot{q}}_{i}<0$.

### 2.2. The Proposed Linear Identification Model

Accurate dynamic models are frequently restricted to identifying important system parameters and compensating for nonlinear terms. Friction, as a primary nonlinear element in robotics, has a significant influence on model accuracy, particularly in the case of velocity reversal and low-speed disturbance, which are difficult to precisely represent. The conventional linear identification approach employs a simplified static friction model that cannot fully capture the dynamic behavior of the friction, such as the non-linearity of viscous friction seen in most HD and the temperature dependency of friction. Especially, augmented dynamic parameters in Equation (4) are highly affected by the robot joint temperature conditions. A significant variation can be exhibited in the values of these parameters depending on the temperature (hot or cold) condition of robot joints during the identification process. The experimental results presented in Table A2 in Appendix A show that for the conventional method, a significant variation in the identified dynamic parameters can be observed (highlighted part shows parameters with more than 20% relative change compared to the cold condition for conventional identification method) depending on the robot joint temperature condition during the identification process.

Therefore, to increase the robustness of conventional identification, we proposed a two-step identification procedure. In the first step, parameters of a comprehensive friction model Equation (16), which was reported in our previous work [13], are identified for each robot joint. Using the identified comprehensive friction model parameters, friction torque is computed for the measured trajectory and joint temperature during dynamic parameter identification. After subtracting friction torque from the recorded joints’ torque, a modified conventional linear identification model Equation (6) is used in the second step of the dynamic parameter identification procedure.

Friction torques are considered a sum of estimates and error terms. With a reasonable assumption that the friction error ${\tilde{\tau }}_{s}≜\tau_{s}-{\hat{\tau }}_{s}$ contain Coulomb, viscous, and offset friction contributions; that is, ${\tilde{\tau }}_{s}={\tilde{F}}_{c}\mathrm{sign}(\dot{q})+{\tilde{F}}_{v}\dot{q}+{\tilde{F}}_{o}$, Equation (4) is modified by replacing all friction related parameters with friction error model parameters such as ${\tilde{F}}_{c,i},{\tilde{F}}_{v,i},$ and ${\tilde{F}}_{o,i}$ for i=1,⋯,N.

Thus, the proposed linear identification model is given by

(6) $$\tau_{mf}=Y_{s}\left(q,\dot{q},\ddot{q}\right)\Phi_{p}$$

where $\Phi_{p}={\left[\Phi_{p,1}^{T}\cdots \Phi_{p,N}^{T}\right]}^{T}$ with $\Phi_{p,i}={\left[\Phi_{i}^{T},{\tilde{F}}_{c,i},{\tilde{F}}_{v,i},{\tilde{F}}_{o,i}\right]}^{T}$, and $\tau_{mf}=\tau_{m}-\tau_{f}$. $\tau_{f}$ is the friction torque vector of robot joints computed based on Equation (16). The proposed identification method is more robust, and the variance in the identified parameters can be reduced to 20% or less in relative change as shown in Table A2 in Appendix A.

### 2.3. The Minimum Inertial Parameters

**Figure 1:**
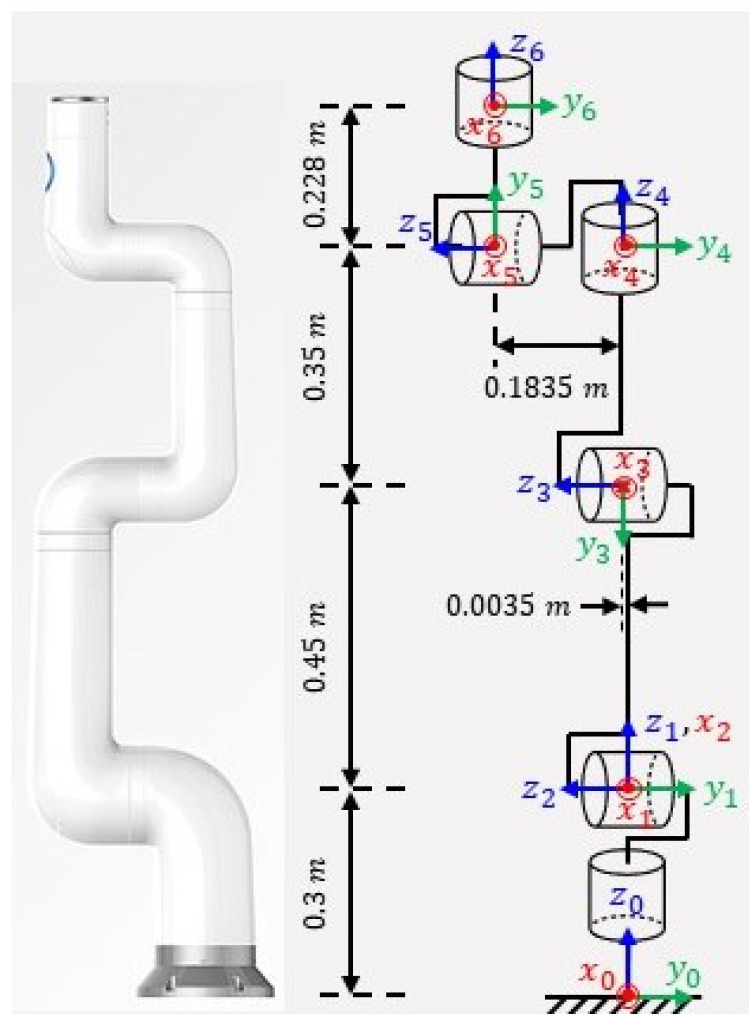
Indy7 robot and its link coordinate system.

Not all 11N inertial parameters of a robot manipulator can be identified; some inertial parameters have no influence on the dynamic model, while others have an effect only in linear combinations. As a result, the set of standard dynamic parameters $\Phi_{s}$ to be identified can be reduced to a minimal set of parameters known as base parameters, $\Phi_{b}\in R^{b\times 1}$, where *b* is the number of base parameters. These parameters can be obtained from the standard inertial parameters by eliminating those which have no effect on the dynamic model and by regrouping some others in linear relations [22,23,24]. Following numerical approach [24], a set of base parameters are computed for the Indy7 robot manipulator with coordinate systems placed as illustrated in Figure 1 and with the modified Denavit-Hartenberg (mDH) parameters in Table 1. The result is presented in Table A1 in Appendix A.

Therefore, the joint torque can be expressed as

(7) $$\tau_{mf}=Y_{B}\left(q,\dot{q},\ddot{q}\right)\Phi_{B}$$

where $Y_{B}\left(q,\dot{q},\ddot{q}\right)\in R^{N\times b}$ is the observation matrix with full rank, and $\Phi_{B}\in R^{(b+3N)\times 1}$ is base parameters,$\Phi_{b}$, augmented with, friction parameters for the conventional case or friction error parameters for the proposed case.

### 2.4. Friction Modeling

In prior work [13], we established a comprehensive friction model that included nonlinear viscous and temperature dependence. A basic overview of the formulation of the comprehensive friction model will be offered here (for further information, please see [13]).

Numerous static and dynamic friction models [9,25,26] have been investigated to characterize friction behavior. Unlike static friction models, which are discontinuous during velocity reversal, the friction phenomena have a nonlinear continuous behavior at velocity zero-crossing. As a result, for a smooth transition and a more accurate representation of the friction phenomenon, it is preferable to adopt a dynamic friction model. A dynamic friction model $\tau_{f}$ in its generic form that takes into account internal state *z* (potentially multiple states), velocity, and temperature-dependent effects can be described by

(8) $$\tau_{f,i}=\delta \left(z_{i}\right)+\zeta \left({\dot{q}}_{i},T_{i}\right),\;i=1,\cdots ,N$$

where $\delta (z_{i})$ is the transient velocity response function, $\zeta ({\dot{q}}_{i},T_{i})$ is the temperature,$T_{i}$, dependent velocity strengthening function including viscous friction. As all consideration, unless otherwise mentioned, is for a single joint, we shall hereafter use a plain symbol without the subscript *i*. Using a first-order differential equation of a generic form, the dynamics of the internal states *z* may be expressed as follows:

(9) $$\frac{dz}{dt}=G\left(z,\dot{q},q\right)$$

with G(·) a general nonlinear function. Since the steady-state friction is a function of relative velocity only ($G(z,\dot{q},q)=0$), the generic form of dynamic friction model Equation (8) converges to a static friction model Equation (10).

(10) $$\tau_{f,s}=g\left(\dot{q}\right)\mathrm{sign}\left(\dot{q}\right)+\zeta \left(\dot{q},T\right)$$

where the generic form of a velocity weakening function (Stribeck effect) $g(\dot{q})$ is given by

(11) $$g\left(\dot{q}\right)=F_{c}+\left(F_{s}-F_{c}\right)e^{-|\dot{q}/v_{s}{|}^{2}}$$

where $F_{c}$ is Coulomb friction, $F_{s}$ is stiction, and $v_{s}$ is Stribeck velocity.

The LuGre friction model [26] is a particular instance of the generic form dynamic friction model illustrated in Equation (8), in which viscous friction is depicted as a linear proportional to relative velocity. Considering that the model is based on the average behavior of the contact between bristles, the model of the average deflection *z* can be given by

(12) $$\frac{dz}{dt}=\dot{q}-\sigma_{0}\frac{|\dot{q}|}{g(\dot{q})}z$$

where, $\sigma_{0}$ is the bristle stiffness. Therefore, the expression for the transient velocity response function $\delta (z_{i})$ becomes

(13) $$\delta \left(z\right)=\sigma_{0}z+\sigma_{1}\dot{z}$$

where $\sigma_{1}$ is the micro-damping coefficient.

#### 2.4.1. Nonlinear Viscous Friction Model

The most typical friction contacts in robotic actuation are the lubricated friction contacts in the main HD. In contrast to the typically employed linear viscous friction, the robot actuators with HD exhibit significantly nonlinear viscous friction. In fact, changes in joint temperature cause a considerable change in viscous friction for the same joint speed. As a result, the viscous friction model is now a function of joint temperature as well as velocity.

#### 2.4.2. Temperature Dependent Viscous Friction Model

At high joint temperatures, friction in HD tends to decrease considerably due to changes in contact point characteristics and lubricating layer thickness. In contrast, at low joint temperatures, the viscosity of the lubricant increases, leading to an increase in viscous friction. Considering such dependencies of viscous friction on temperature, we proposed an exponential model that maps the nonlinear viscous friction behavior over the joint velocity range, Equation (14). To account for temperature dependency, the parameters of the nonlinear viscous friction model are further parameterized as a function of joint temperature Equation (15).

(14) $$f_{v}\left(\dot{q},T\right)=\sigma_{2}\left(T\right)\left(1-e^{-|\sigma_{3}\left(T\right)\dot{q}{|}^{\sigma_{4}\left(T\right)}}\right)$$

where

(15) $$\sigma_{j}\left(T\right)=\sigma_{jo}+\sigma_{jT}T\;\mathrm{for}\;j=2,3,4$$

#### 2.4.3. Comprehensive Friction Model

Following a thorough analysis of the nonlinear viscous and temperature dependencies, a comprehensive friction model is developed. Taking Equations (13) and (14) into account, a complete friction model can be developed as follows:

(16) $$\tau_{f}\left(\dot{q},T\right)=\sigma_{0}z+\sigma_{1}\dot{z}+f_{v}\left(\dot{q},T\right)$$

## 3. Identification

**Figure 2:**
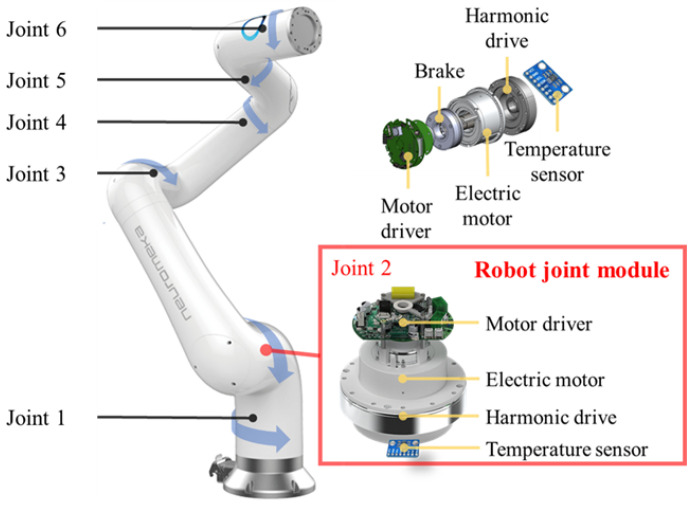
Indy7 collaborative robot manipulator utilized for experimental verification, along with an exploded layout of its joint module, which includes an electric motor, harmonic drive, motor driver, encoder, and temperature sensor.

This section presents the experimental setup, identification procedure, and results. The experiment is carried out using a 6-DOF robot manipulator (Neuromeka, Indy7: 7-kg payload, 28-kg weight, 28-μm repeatability) (Figure 2).

The system is actuated using a brushless DC motor with HD gear transmission. A 16-bit multi-turn absolute encoder measures each angular position. The joint torque is determined by multiplying the measured motor current by the torque constant of the motor. For each joint, a temperature sensor of type MCP9808 is placed on the housing surface of the HD in order to acquire the joint temperature. All data are logged at a sampling frequency of 4 kHz. Data is sampled at times $t\left(k\right)=kT_{s},k=1,2,\cdots ,M$, where $T_{s}=0.25\;\mathrm{ms}$ is the sampling period.

**Figure 3:**
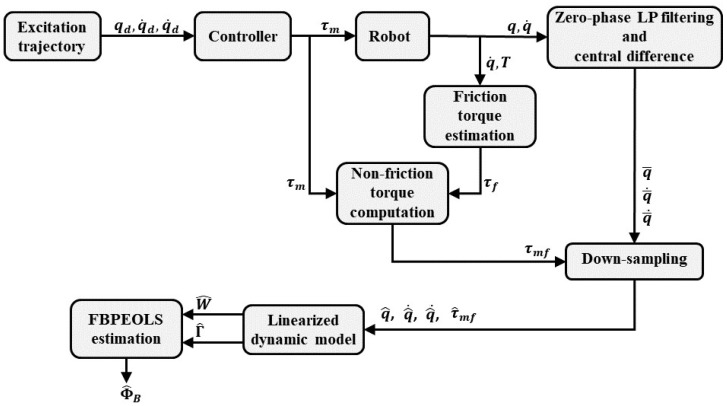
The proposed dynamic parameters identification process.

The identification procedure is illustrated schematically in Figure 3. The identification procedure can generally be divided into two steps. In general, the identification procedure can be classified into two steps. First, the friction of each joint is identified. The parameters of the comprehensive friction model in Equation (16) are identified. After identifying the parameters of the comprehensive friction model, the next step is to identify the parameters of the robot’s dynamic model. Estimated friction torque based on a comprehensive friction model is subtracted from measured torque during robot dynamic model parameter identification $\tau_{m}$. This non-friction torque $\tau_{mf}$ will be used to identify the robot’s dynamic model parameters based on the proposed identification method. These identification procedures will be described in full in the sections that follow.

### 3.1. Friction Identification

#### 3.1.1. Static Model Identification

An extensive experimental investigation was carried out to gain insights and distinguish relationships in the velocity and temperature during the identification of friction model parameters. To begin, the joint is rotated back and forth at various steady rates. We may presume that the centrifugal and inertial torques are negligible since only a single joint is moving at a time and data is collected for constant velocity region. As a result, the applied torque may be defined as the total of gravity-induced and friction torques, Equation (17),

(17) $$G_{i}\left(q_{i}\right)+\tau_{f,i}=\tau_{m,i}\;i=1,\cdots ,N.$$

As all consideration, unless otherwise mentioned, is for a single joint, we shall hereafter use a plain symbol without the subscript *i*. An estimate of friction that is independent of direction can be obtained by taking into account forward and reverse motions for a speed level of $\dot{q}$. Since gravity-induced torque is directional, it may be canceled out, and the friction torque can be calculated as follows,

(18) $$\tau_{f}=\left(\tau_{m}^{+}-\tau_{m}^{-}\right)/2$$

where $\tau_{m}^{+}$ and $\tau_{m}^{-}$ are the resulting torques when the joint is moved forward and backward, respectively. The procedure can be repeated for several $\dot{q}$’s and a friction curve can be drawn, which contains steady-state friction values. To observe temperature dependencies, the above procedure was repeated for different constant velocity points covering the considered velocity range over different constant joint temperatures. The temperature deviation during every individual experiment is maintained at ≤1 °C, which is assumed as an acceptable uncertainty.

**Figure 4:**
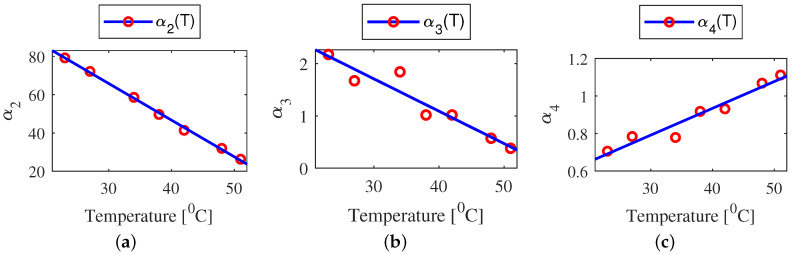
The variation of viscous friction model parameters (**a**) $\alpha_{2}\left(T\right),\left(b\right)\alpha_{3}\left(T\right),\left(c\right)\;\alpha_{4}\left(T\right)$ for joint one. The model parameters change with respect to the joint temperature are indicated with markers, and *Model Fit* corresponding to Equation (15) are indicated with a solid line.

The collected data are used to identify different temperature-independent friction curves Equation (19)

(19) $$g\left(\dot{q}\right)+f_{v}\left(\dot{q}\right)=F_{c}+\left(F_{s}-F_{c}\right)e^{-|\dot{q}/v_{s}{|}^{2}}+\sigma_{2}\left(1-e^{-|\sigma_{3}\dot{q}{|}^{\sigma_{4}}}\right)$$

where $f_{v}\left(\dot{q}\right)=f_{v}\left(\dot{q},T=const\right)$ is viscous friction at constant temperature case. Then, further parameterized viscous friction model parameters, Equation (15), were identified using the estimated parameters in Equation (19) for different joint temperatures (see Figure 4a–c). It should be noted that the temperature dependence of parameters of the velocity weakening function, $g(\dot{q})$, is neglected due to negligible variations with temperature change.

**Figure 5:**
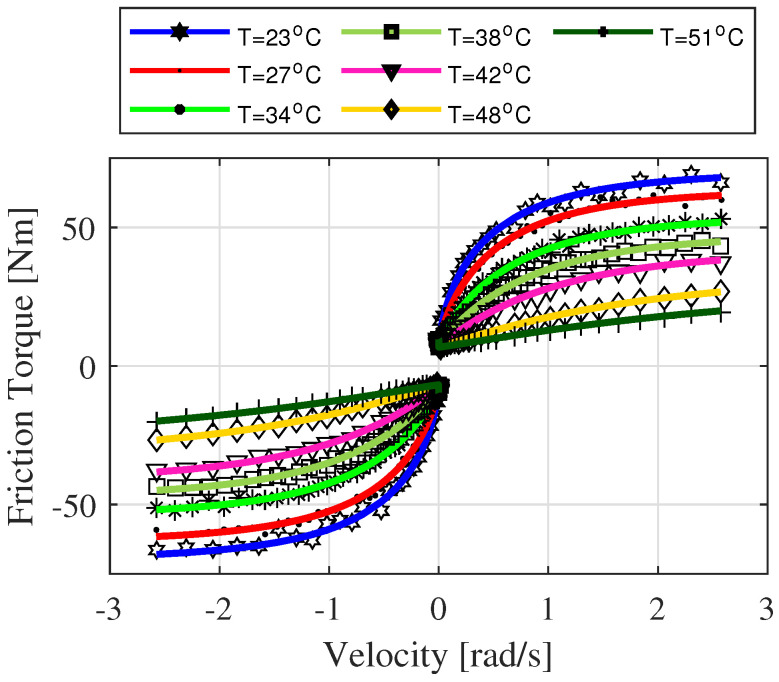
The dependency of friction on velocity and temperature for joint one. Static friction: experimental data (markers) and *Model Fit* (solid lines) corresponding to Equation (19) for different joint temperature values.

Because the remaining joints of the robot show a similar pattern, only the result for joint one is shown in Figure 4 and Figure 5. Figure 5 depicts the total result of the proposed velocity-temperature model Equation (14). For a more detailed identification procedure refer to [13].

#### 3.1.2. Dynamic Model Identification

To identify dynamic friction model parameters, $\sigma_{0}$ and $\sigma_{1}$, the approach used in [27] was addapted in this work. In this experiment, parameters for the dynamic friction model, $\sigma_{0}$ and $\sigma_{1}$, were determined by adapting a method from [27]. Open-loop experiments were conducted with a sinusoidal torque input, and the resulting data were used to estimate dynamic parameters using a pattern search solver in the MATLAB optimization toolbox with a default option. The results of the identified parameters of the dynamic friction model are shown in Table 2.

### 3.2. Inertia Parameter Identification

#### 3.2.1. Joint Position, Velocity, and Acceleration Estimation

Because the noise in recorded data is the primary source of errors in parameter identification, signal processing is essential for ensuring the quality of the measured data. In our experiment, the measured trajectory data $q,\dot{q}$ are filtered by a 4th order Butterworth filter in both forward and reverse directions to eliminate lag of the filtered trajectories $\bar{q},\dot{\bar{q}}$. The value of angular acceleration is calculated by differential equations since it cannot be measured directly from the Indy7 collaborative robot system. To avoid phase shifts in the differential calculation, angular acceleration $\ddot{\bar{q}}$, is computed through a central difference procedure.

Since the sampling frequency is much higher than the frequencies of interest in the dynamics, the data is decimated/down-sampled to reduce the required computational resources. In this experiment, a donwsampling factor of $0.8f_{s}/\left(4f_{dyn}\right)=80$ were used, where $f_{s}=4\;\mathrm{kHz}$ and $f_{dyn}=10\;\mathrm{Hz}$ [28]; that is, every 80th sample is used for parameter estimation. Thus, to remove information-free samples, a down-sampling is conducted on the filtered trajectories $\bar{q},\dot{\bar{q}}$ and $\ddot{\bar{q}}$.

#### 3.2.2. Torque Computation

First, the samples k=1,⋯,M are ordered in the measurement vector $\Gamma_{i}$ and observation matrix $W_{i}$ for each joint i=1,⋯,N individually; that is

(20) $$\begin{array}{cc} \Gamma_{i} & ={\left[\tau_{mf,i,1}\cdots \tau_{mf,i,k}\cdots \tau_{mf,i,M}\right]}^{T},\\ W_{i} & =[W_{i,1}\cdots W_{i,k}\cdots W_{i,M}]\\ W_{i,k} & =Y_{b,i}\left({\hat{q}}_{k},{\dot{\hat{q}}}_{k},{\ddot{\hat{q}}}_{k}\right) \end{array}$$

with $Y_{b,i}\left({\bar{q}}_{k},{\dot{\bar{q}}}_{k},{\ddot{\bar{q}}}_{k}\right)$ being the *i*th row of the regressor evaluated in the *k*th sample of the filtered trajectory. The filtered data are ordered joint-wise in the measurement vector and observation matrix as

(21) $$\begin{array}{cc} \Gamma & ={\left[\Gamma_{1}^{T}\cdots \Gamma_{i}^{T}\cdots \Gamma_{N}^{T}\right]}^{T}\;\in R^{N.M} \end{array}$$

(22) $$\begin{array}{cc} W & ={\left[W_{1}^{T}\cdots W_{i}^{T}\cdots W_{N}^{T}\right]}^{T}\;\in R^{N.M\times (b+3N)} \end{array}$$

Then, the measurement vector Γ is sampled down with the same downsampling factor $0.8f_{s}/\left(4f_{dyn}\right)=80$, and the observation matrix W is ordered with $Y_{b,i}\left({\hat{q}}_{k},{\dot{\hat{q}}}_{k},{\ddot{\hat{q}}}_{k}\right)$ being the *i*th row of the regressor evaluated in the *k*th sample of the filtered and downsampled trajectory instead. Finally, using the downsampled measurement vector, $\hat{\Gamma }$, and the observation matrix formulated with the corresponding filtered and downsampled trajectories, $\hat{W}$, the base parameters are estimated by solving the weighted least square (WLS) problem:

(23) $$\begin{array}{c} \begin{array}{cc} {\hat{\Phi }}_{B} & =\arg\min_{\Phi_{B}}\left|\right|{\hat{W}}^{T}\Lambda \left(\hat{\Gamma }-\hat{W}\Phi_{B}\right){\left|\right|}^{2}\\ \; & ={\left({\hat{W}}^{T}\Lambda \hat{W}\right)}^{-1}{\hat{W}}^{T}\Lambda \hat{\Gamma } \end{array} \end{array}$$

where Λ is a weight matrix given by [29]:

(24) $$\begin{array}{cc} \Lambda & =diag(D),\\ D & =[D_{1}\;\cdots \;D_{i}\;\cdots D_{N}],\\ D_{i} & =\left[\frac{1}{{\hat{\sigma }}_{i,1}}\;\cdots \;\frac{1}{{\hat{\sigma }}_{i,j}}\;\cdots \frac{1}{{\hat{\sigma }}_{i,b_{i}}}\right],\;j=1,\cdots ,b_{i}\\ {\hat{\sigma }}_{i,j}^{2} & =\frac{\left|\right|\tau_{i}-Y_{B,i}{\hat{\Phi }}_{B,i}{\left|\right|}^{2}}{M-b_{i}} \end{array}$$

with $b_{i}$ being the number of base parameters related to link *i*.

#### 3.2.3. Excitation Trajectory Design

**Figure 6:**
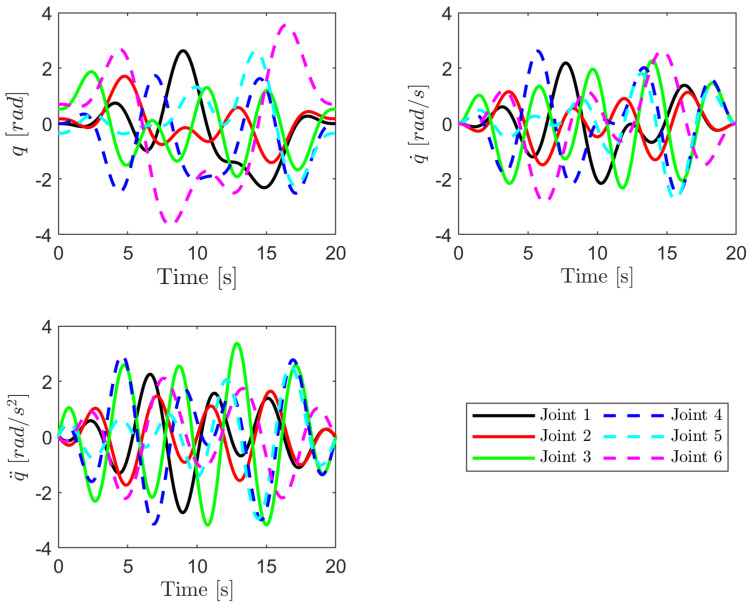
Optimized robot excitation trajectories.

The goal of the excitation trajectory optimization is to find a trajectory that can sufficiently excite the identified dynamic parameters. The trajectory used for parameter estimation has been designed to optimize the condition number of the regressor matrix, W. Typically, a finite Fourier series method Equation (25), which suppresses measurement noise, is used to generate periodic trajectories [30]. Each *k*th joint trajectory is defined as a function of time *t* by

(25) $$\begin{array}{cc} q_{i}\left(t\right) & =\sum_{l=1}^{L}(\frac{a_{l}^{i}}{\omega_{f}l}sin\left(\omega_{f}lt\right)-\frac{b_{l}^{i}}{\omega_{f}l}cos\left(\omega_{f}lt\right))+q_{io}\\ {\dot{q}}_{i}\left(t\right) & =\sum_{l=1}^{L}(a_{l}^{i}cos\left(\omega_{f}lt\right)+b_{l}^{i}sin\left(\omega_{f}lt\right))\\ {\ddot{q}}_{i}\left(t\right) & =\omega_{f}\sum_{l=1}^{L}l(b_{l}^{i}cos\left(\omega_{f}lt\right)-a_{l}^{i}sin\left(\omega_{f}lt\right)) \end{array}$$

where in this experiment $w_{f}=0.1\pi$ is the fundamental frequency of the Fourier series, $q_{io}$ is the offset value of the position trajectory and L=5 is the number of harmonics. The optimal excitation trajectory is generated, which yields in a condition number of cond(W)=80.25, and the result is shown in Figure 6 for a 20 s long trajectories.

### 3.3. Physical Feasibility of the Dynamic Parameters

Physical constraints on real robot dynamic parameters such as mass and inertia tensors must be considered to obtain meaningful estimations. To this end, the estimated dynamic parameters ${\hat{\Phi }}_{B}$ might be possibly physically infeasible (e.g., a negative link mass or not positive definite inertia tensor), and this can be caused, for instance, by modeling error or by noisy measurements. Over the last two decades, extensive study in robotics has been conducted on the idea of imposing physical feasibility as part of a parameter identification process. The physical feasibility of parameters is formulated as constraints on its mass $m_{i}$, viscous-Coulomb friction $F_{v,i},F_{c,i}$, joint module inertia $I_{ai}$, and link’s center-of-mass (CoM) $I_{i}$ parameters as follows [31,32,33]:

(26) $$\forall \;link\;i:\left\{\begin{array}{c} m_{i}>0,\;F_{v,i}>0,\;F_{c,i}>0,I_{ai}>0\\ I_{i}≻0 \end{array}\right.$$

where

$$I_{i}=\left(\begin{array}{ccc} Ixx_{i} & Ixy_{i} & Ixz_{i}\\ Ixy_{i} & Iyy_{i} & Iyz_{i}\\ Ixz_{i} & Iyz_{i} & Izz_{i} \end{array}\right)$$

Since inertial parameters are typically calculated using linear regression methods, the inertia tensor is computed about the link frame *i*, $L_{i}$,

(27) $$L_{i}=\left(\begin{array}{ccc} Lxx_{i} & Lxy_{i} & Lxz_{i}\\ Lxy_{i} & Lyy_{i} & Lyz_{i}\\ Lxz_{i} & Lyz_{i} & Lzz_{i} \end{array}\right)$$

has to be used instead of $I_{i}$ [18]. Therefore, the set of all physical feasibility constraints for each link *i* given by Equation (26) can then be written as

(28) $$\forall \;link\;i:\left\{\begin{array}{c} m_{i}>0,\;F_{v,i}>0,\;F_{c,i}>0,I_{ai}>0\\ L_{i}-\frac{1}{m_{i}}S{\left(l_{i}\right)}^{T}S\left(l_{i}\right)≻0 \end{array}\right.$$

where S(·) is the skew-symmetric matrix operator and $l_{i}$ is first moment-of-inertia vector,

$$S\left(r_{i}\right)=\left(\begin{array}{ccc} 0 & -z_{i} & y_{i}\\ z_{i} & 0 & -x_{i}\\ -y_{i} & x_{i} & 0 \end{array}\right),\;l_{i}=m_{i}r_{i}=\left(\begin{array}{c} mx_{i}\\ my_{i}\\ mz_{i} \end{array}\right)$$

with $r_{i}={\left[x_{i}\;y_{i}\;z_{i}\right]}^{T}$ is the center of mass relative to the link frame *i* (see [33]). Observing that the second inequality of Equation (28) is in fact the Schur complement of a matrix $B_{L,i}\left({\bar{\Phi }}_{s}\right)$ allows rewriting Equation (28) as

(29) $$B_{i}\left({\bar{\Phi }}_{s}\right)=\left(\begin{array}{cc} B_{Li}\left({\bar{\Phi }}_{s}\right) & 0_{6\times 3}\\ 0_{3\times 6} & B_{Ai}\left({\bar{\Phi }}_{s}\right) \end{array}\right)≻0$$

where

$$B_{Li}\left({\bar{\Phi }}_{s}\right)=\left(\begin{array}{cc} L_{i} & S{\left(l_{i}\right)}^{T}\\ S(l_{i}) & m_{i}1_{3\times 3} \end{array}\right),\;B_{Ai}\left({\bar{\Phi }}_{s}\right)=\left(\begin{array}{ccc} F_{v,i} & 0 & 0\\ 0 & F_{c,i} & 0\\ 0 & 0 & I_{ai} \end{array}\right)$$

where $0_{n\times m}$, $1_{3\times 3}$ respectively denote the n×m zero, and 3×3 identity matrices. For a robot with *N* links, the feasibility condition can be expressed as

(30) $$B({\bar{\Phi }}_{s})≻0$$

where $B({\bar{\Phi }}_{s})$ is a single block-diagonal matrix given by

(31) $$B\left({\bar{\Phi }}_{s}\right)=\left(\begin{array}{cccc} B_{1}\left({\bar{\Phi }}_{1}\right) & 0 & \cdots & 0\\ 0 & B_{2}\left({\bar{\Phi }}_{2}\right) & \cdots & 0\\ \vdots & \vdots & \ddots & \vdots \\ 0 & 0 & \cdots & B_{N}\left({\bar{\Phi }}_{N}\right) \end{array}\right)$$

After applying a bijective affine transformation to a convex spectrahedron set $B=\{{\bar{\Phi }}_{s}\in R^{n}:B\left({\bar{\Phi }}_{s}\right)≻0\}$, a new physically feasible extended base parameter set, $B_{B}$, can be defined as

(32) $$B_{B}=\left\{\Phi_{B}\in R^{n}:B_{B}\left(\Phi_{B},{\bar{\Phi }}_{s,d}\right)≻0\right\}$$

where n=13N, and ${\bar{\Phi }}_{s,d}$ is a reordered dynamic parameter according to linearly independent columns of the regressor matrix (see [33] for details). Finally, a method for feasible base parameter estimation with ordinary least square (FBPEOLS) formulated in [33] is used to compute the physically feasible base parameter vector ${\hat{\Phi }}_{B}$ which minimizes the sum of squared residuals,

(33) $$\begin{array}{cc} \min_{\left(u,\Phi_{B},{\bar{\Phi }}_{s,d}\right)} & \;u\\ \mathrm{subject}\mathrm{to}\; & F(u,\Phi_{B},{\bar{\Phi }}_{s,d})⪰0 \end{array}$$

where

$$\begin{array}{cc} F(u,\Phi_{B},{\bar{\Phi }}_{s,d}) & =\left(\begin{array}{cc} U(u,\Phi_{B},) & 0\\ 0 & B_{B}\left(\Phi_{B},{\bar{\Phi }}_{s,d}\right) \end{array}\right) \end{array}$$

### 3.4. Model Quality Metric

During verification experiments, the confidence of the dynamic model with identified dynamic parameters is verified using specific trajectories. A root-mean-square error (RMSE) is used to evaluate the model quality numerically:

(34) $$RMSE_{i}=\sqrt{\frac{1}{M}\sum_{k=1}^{M}{\left(\tau_{mf,i,k}-{\hat{\tau }}_{mf,i,k}\right)}^{2}}$$

for joint *i*.

### 3.5. Results

For identification of base parameters, a persistent excitation trajectory as depicted in Figure 6 is generated. The observation matrix $\hat{W}$ is constructed with the measured, filtered, and decimated joint trajectories. The measurement vector $\hat{\Gamma }$ is also constructed with the measured and decimated non-friction torques, ${\hat{\tau }}_{mf}$. For comparison purposes, base parameters are identified using the conventional method, where the measurement vector $\hat{\Gamma }$ is constructed with the measured and decimated torques, ${\hat{\tau }}_{m}$. Then, a method for feasible base parameter estimation with OLS, Equation (33) is applied and the physically feasible base parameters are computed for both conventional (Equation (5)) and proposed (Equation (6)) methods.

The effectiveness of the presented identification method is demonstrated by comparing the accuracy of the dynamic model with parameters obtained using the proposed linear identification model Equation (6) to the accuracy of the dynamic model with parameters obtained using a conventional linear identification model Equation (5). Since we observed significant friction torque variations between cases of low joint temperature and cases of high joint temperature, the accuracy of the dynamic model is evaluated for two of these temperature extremes: (1) at the low joint temperature (19∼22 °C) and (2) at the high joint temperature (40∼46 °C). The scenario in which the joint temperature is low is when the robot begins operation, whereas the condition in which the joint temperature is high is when the robot experiences a thermal transient after a lengthy period of operation. The dynamic model parameters are identified for moderate robot joint temperature (29∼33 °C) condition.

**Figure 7:**
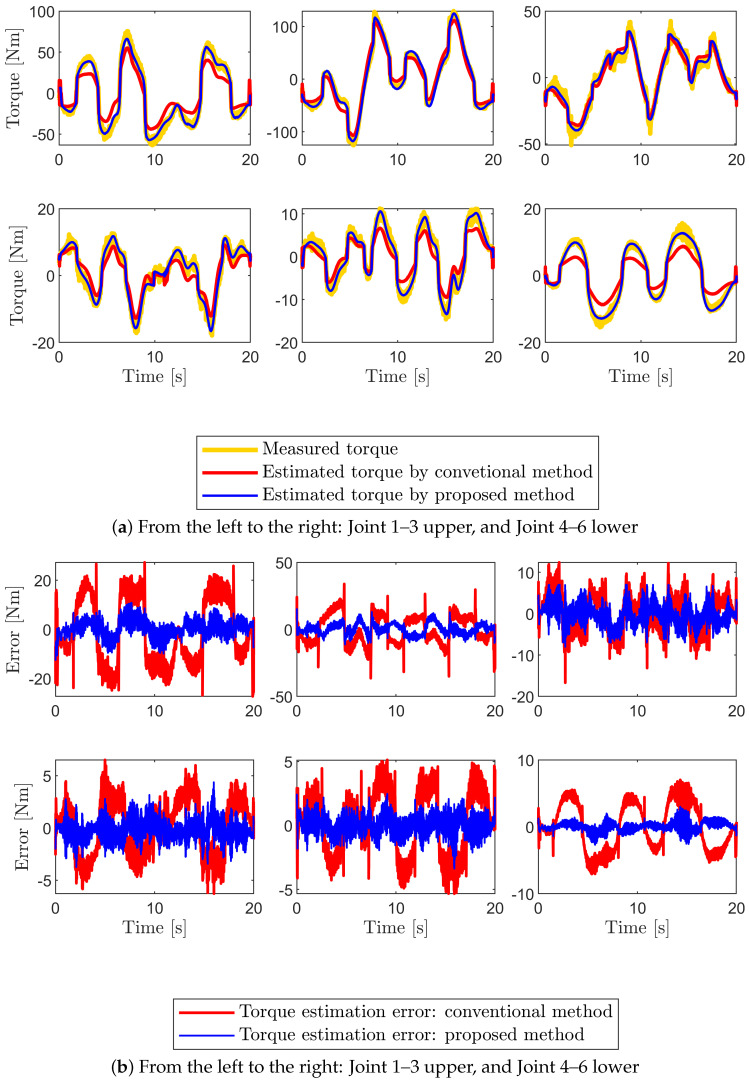
(**a**) Estimated torque comparison between measured torque (yellow lines) and the predicted torque using the identified base parameters from the proposed method (blue line) and the conventional method (red line) for the excitation trajectories. (**b**) The corresponding torque estimation error. The experiment is performed at the low joint temperature (19∼22 °C).

**Figure 8:**
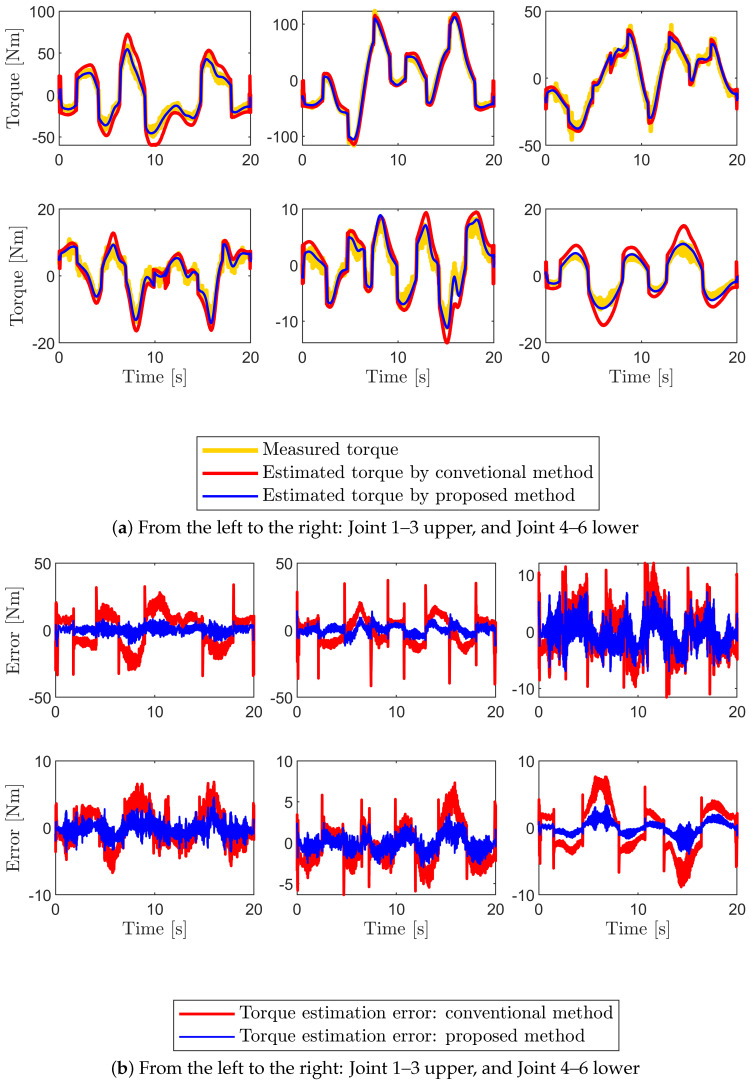
(**a**) Estimated torque comparison between measured torque (yellow lines) and the predicted torque using the identified base parameters from the proposed method (blue line) and the conventional method (red line) for the excitation trajectories. (**b**) The corresponding torque estimation error. The experiment is performed at the high joint temperature (40∼46 °C).

To evaluate the accuracy of the estimated parameters, predicted and measured torques are compared. The experimental findings demonstrate that the estimated joint torque by the identified parameters using the proposed method can be predicted well when compared to the estimated torque using the conventional method as shown in Figure 7 and Figure 8. For instance, from Figure 7b we can observe that the model accuracy improves 74.6% from RMSE of 13.44 Nm to that of 3.42 Nm for joint one at the low joint temperature condition. The model accuracy also improves 82.9% from RMSE of 15.95 Nm to that of 2.72 Nm for joint one at the high joint temperature condition as shown in Figure 8b. In both temperature conditions, the model accuracy is evaluated using the same excitation trajectories employed during parameter identification (Figure 6). To confirm that the obtained dynamic model is accurate and reliable, new excitation trajectories are generated and the accuracy of the model is evaluated in the subsequent section.

### 3.6. Validation

To validate the results of robot dynamic parameter identification and the relevance of the proposed method, a new robot trajectory different from the trajectory used for identification is generated. Experiments of validation are conducted for three separate reference trajectories, Trajectory A, Trajectory B, and Trajectory C, with respective condition numbers of 70.3, 74.9, and 104.5. A comparison is made between the torques obtained by the dynamic model with parameters obtained using the proposed linear identification model, Equation (6), and the torques obtained by the dynamic model with parameters obtained using the conventional linear identification model, Equation (5). To further validate the accuracy of the estimated parameters, the RMSE of residuals between the measured torque and the estimated torque obtained from the identification model is introduced. The relative improvement in RMSE between the conventional and proposed methods is also examined. The dynamic model’s accuracy was validated for two extreme joint temperature scenarios (low and high joint temperature). Table 3 and Table 4 show the results of the root mean square error (RMSE) and relative improvement analyses for the low and high joint temperature scenarios, respectively. According to the results, the proposed identification approach has the potential to achieve a relative improvement that ranges from 38% to 78.37% when compared to the conventional one for the low joint temperature scenario. For the scenario where the joint temperature is high, it is possible to achieve a relative improvement of 15.05% to 61.64%.

To sum up, the dynamic model obtained based on the suggested method can reduce torque prediction error by up to 78.37% and 61.64% in the low and high joint temperature cases, respectively. This proves that the proposed parameter estimation method is effective.

## 4. Discussions

Experimental results indicate that identifying the dynamic parameters of the collaborative robot manipulator using the proposed identification approach improves the accuracy of the robot dynamic model. It is important to note that the torque prediction errors for the first and second joints are higher than those for the other joints. Due to their high self-weight, the first and second joint modules include a larger HD with a gear ratio of 121:1 and a motor power rating of 500 W. This causes these two joints to have greater joint friction than the others. For a conventional linear identification model, ignoring such a significant joint nonlinearity due to friction leads to a higher torque prediction error for the first two joints in particular. However, by integrating a more realistic friction model and adopting the proposed identification strategy during robot dynamic model parameter estimation, the proposed method can mitigate these problems and significantly reduce torque prediction errors.

During robot dynamic model parameter identification, considering a realistic friction model that reflects the variance due to joint temperature changes over extended operation increases the model’s accuracy. From the experimental data, it is obvious that there is a considerable variation in joint friction during robot startup and after the robot’s thermal steady state is attained. Since linearity in the parameters might be lost during long time periods of robot operation, such fluctuation in friction owing to thermal factors leads to model mismatch. In contrast to friction characteristics, robot dynamic parameters should be temperature-independent.

Nevertheless, ignoring highly nonlinear and temperature-dependent friction during the identification procedure, which leads to model mismatches over the extended operation, may lead to an incorrect estimation of the robot’s dynamic model parameters. This could lead to a less accurate robot dynamic model. As seen in the preceding section, experimental results also indicate that using a more realistic friction model during identification can increase the accuracy of the robot’s dynamic model. The proposed method could be limited by the accessibility of robot joint temperature data. In this study, we assumed that each joint has a temperature sensor. Future work may include the development of sensorless joint temperature estimation for collaborative robot manipulators lacking a temperature sensor.

## 5. Conclusions

In this study, the problem of identifying the dynamic parameters of collaborative robots with a more realistic friction model is examined. The primary source of nonlinear joint dynamics, such as friction, in a collaborative robot manipulator, is a strain-wave transmission mechanism, such as the HD, which is commonly employed in collaborative robot manipulators due to its high-quality attributes of loading capacity and lightweight. Such nonlinear joint dynamics have a significant impact on the accuracy of the robot’s dynamic parameters if not taken into account during the identification procedure. Moreover, the lubricated friction contacts in HD are greatly impacted by the variation in joint temperature, resulting in a noticeable change in friction due to the variation in joint temperature. As a result, the primary objective of this research is to incorporate nonlinear joint dynamics into the identification process of the robot’s dynamic model parameters. Since the current robot dynamic model parameter identification method is based on the assumption of the dynamic model’s linearity, it is hard to directly incorporate a nonlinear friction model. Therefore, we suggest a strategy consisting of two steps, the first of which is to identify a comprehensive friction model. This friction model is utilized to estimate joint friction as a function of joint velocity and temperature. Then, we proposed an identification technique in which a non-friction torque would be utilized in the robot dynamic parameter identification step. This would replace the practice of utilizing measured joint torque, which would have included friction.

Using the proposed identification approach, the parameters of the Indy7 dynamic model were obtained, and the torque prediction accuracy of the robot’s dynamic model was assessed. Experiments of validation demonstrated that the torque estimation of the dynamic model obtained using identified parameters based on the proposed method can accurately represent the robot’s dynamic characteristics. The results of the experiments that were done with the validation trajectories show that the proposed method is capable of achieving a reduction in torque prediction error of up to 78.37% in the low joint temperature case and up to 61.64% in the high joint temperature case This proves that the proposed parameter estimation method is effective.

Future work will be expanded to include payload estimation, and use the derived dynamic model for applications such as collision detection and model-based robot controller design. In addition, as a future study, the proposed method will be contrasted and analyzed with more advanced identification algorithms that take joint friction into account.

## Figures and Tables

**Table 1:** Modified Denavit-Hartenberg parameters of Indy7 robot.

Link i	$a_{i-1}\left(m\right)$	$\alpha_{i-1}\left(\mathrm{rad}\right)$	$d_{i}\left(m\right)$	$\theta_{i}\left(\mathrm{rad}\right)$
1	0	0	0.3	$q_{1}$
2	0	π/2	0	$q_{2}+\pi /2$
3	0.45	0	0.0035	$q_{3}+\pi /2$
4	0	π/2	0.35	$q_{4}+\pi$
5	0	π/2	0.1835	$q_{5}$
6	0	−π/2	0.228	$q_{6}$

**Table 2:** Estimated parameters of the dynamic friction model.

	Joint-1	Joint-2	Joint-3	Joint-4	Joint-5	Joint-6
$\sigma_{0}$	16,804	12,930	1820	1649	1106	985
$\sigma_{1}$	270	233	105	81	72	68

**Table 3:** RMSE of the dynamic models: with parameters obtained using conventional and proposed linear identification models. Improvements in RMSE relative to the dynamic model with parameters obtained using the conventional linear identification model. The validation experiment is performed at the lower joint temperature (19∼22 °C).

Joint No.	Trajectory								
	A			B			C		
	RMSE Conventional	RMSE Proposed	Relative Improvement	RMSE Conventional	RMSE Proposed	Relative Improvement	RMSE Conventional	RMSE Proposed	Relative Improvement
1	12.01	2.60	78.37%	10.45	2.47	76.31%	13.79	3.58	74.04%
2	13.28	3.85	71.00%	11.35	3.42	69.90%	9.39	3.79	59.65%
3	3.27	1.90	41.73%	2.90	1.80	38.0%	3.31	1.97	40.62%
4	1.60	0.93	42.05%	1.73	0.58	66.39%	2.10	0.88	58.0%
5	2.25	0.96	57.39%	1.52	0.80	47.14%	2.05	0.94	53.93%
6	3.64	1.09	70.05%	3.52	1.01	71.22%	3.25	0.97	70.12%

**Table 4:** RMSE of the dynamic models: with parameters obtained using conventional and proposed linear identification models. Improvements in RMSE relative to the dynamic model with parameters obtained using the conventional linear identification model. The validation experiment is performed at the higher joint temperature (40∼46 °C).

Joint No.	Trajectory								
	A			B			C		
	RMSE Conventional	RMSE Proposed	Relative Improvement	RMSE Conventional	RMSE Proposed	Relative Improvement	RMSE Conventional	RMSE Proposed	Relative Improvement
1	6.70	2.57	61.64%	6.51	2.91	55.33%	5.85	2.81	52.00%
2	9.25	4.49	51.46%	8.31	4.22	49.22%	5.81	4.38	24.53%
3	3.58	1.99	44.35%	2.73	1.73	36.68%	2.21	1.87	15.05%
4	1.55	0.80	48.35%	1.50	0.58	61.30%	1.11	0.735	33.58%
5	1.21	0.58	52.31%	1.85	0.75	59.49%	0.897	0.574	36.07%
6	1.41	0.66	53.08%	1.55	0.75	51.71%	0.91	0.50	44.75%

## Data Availability

Not applicable.

## Appendix A

### Appendix A.1. Base Parameters

**Table A1:** Base parameters of Indy7.

$\Phi_{B}$	Corresponding Linear Combination
$\Phi_{B1}$	${\mathrm{Ia}}_{1}+{\mathrm{Iyy}}_{2}+{\mathrm{Iyy}}_{3}+{\mathrm{Izz}}_{1}+0.2025\;m_{3}+0.2025\;m_{4}+0.2025\;m_{5}+0.2025\;m_{6}+0.0070\;{\mathrm{mz}}_{3}$
$\Phi_{B2}$	${\mathrm{Fc}}_{1}$
$\Phi_{B3}$	${\mathrm{Fv}}_{1}$
$\Phi_{B4}$	${\mathrm{Fo}}_{1}$
$\Phi_{B5}$	${\mathrm{Ixx}}_{2}-{\mathrm{Iyy}}_{2}-0.2025\;m_{3}-0.2025\;m_{4}-0.2025\;m_{5}-0.2025\;m_{6}$
$\Phi_{B6}$	${\mathrm{Ixy}}_{2}$
$\Phi_{B7}$	${\mathrm{Ixz}}_{2}-0.0016\;m_{3}-0.0016\;m_{4}-0.0016\;m_{5}-0.0016\;m_{6}-0.4500\;{\mathrm{mz}}_{3}$
$\Phi_{B8}$	${\mathrm{Iyz}}_{2}$
$\Phi_{B9}$	${\mathrm{Ia}}_{2}+{\mathrm{Izz}}_{2}+0.2025\;m_{3}+0.2025\;m_{4}+0.2025\;m_{5}+0.2025\;m_{6}$
$\Phi_{B10}$	$0.4500\;m_{3}+0.4500\;m_{4}+0.4500\;m_{5}+0.4500\;m_{6}+{\mathrm{mx}}_{2}$
$\Phi_{B11}$	${\mathrm{my}}_{2}$
$\Phi_{B12}$	${\mathrm{Fc}}_{2}$
$\Phi_{B13}$	${\mathrm{Fv}}_{2}$
$\Phi_{B14}$	${\mathrm{Fo}}_{2}$
$\Phi_{B15}$	${\mathrm{Ixx}}_{3}-{\mathrm{Iyy}}_{3}+{\mathrm{Iyy}}_{4}+0.1225\;m_{4}+0.1225\;m_{5}+0.1225\;m_{6}+0.7000\;{\mathrm{mz}}_{4}$
$\Phi_{B16}$	${\mathrm{Ixy}}_{3}$
$\Phi_{B17}$	${\mathrm{Ixz}}_{3}$
$\Phi_{B18}$	${\mathrm{Iyz}}_{3}$
$\Phi_{B19}$	${\mathrm{Iyy}}_{4}+{\mathrm{Izz}}_{3}+0.1225\;m_{4}+0.1225\;m_{5}+0.1225\;m_{6}+0.7000\;{\mathrm{mz}}_{4}$
$\Phi_{B20}$	${\mathrm{mx}}_{3}$
$\Phi_{B21}$	${\mathrm{my}}_{3}-0.3500\;m_{5}-0.3500\;m_{6}-0.3500\;m_{4}-{\mathrm{mz}}_{4}$
$\Phi_{B22}$	${\mathrm{Ia}}_{3}$
$\Phi_{B23}$	${\mathrm{Fc}}_{3}$
$\Phi_{B24}$	${\mathrm{Fv}}_{3}$
$\Phi_{B25}$	${\mathrm{Fo}}_{3}$
$\Phi_{B26}$	${\mathrm{Ixx}}_{4}-{\mathrm{Iyy}}_{4}+{\mathrm{Iyy}}_{5}+0.0337\;m_{5}+0.0337\;m_{6}+0.3670\;{\mathrm{mz}}_{5}$
$\Phi_{B27}$	${\mathrm{Ixy}}_{4}$
$\Phi_{B28}$	${\mathrm{Ixz}}_{4}$
$\Phi_{B29}$	${\mathrm{Iyz}}_{4}$
$\Phi_{B30}$	${\mathrm{Iyy}}_{5}+{\mathrm{Izz}}_{4}+0.0337\;m_{5}+0.0337\;m_{6}+0.3670\;{\mathrm{mz}}_{5}$
$\Phi_{B31}$	${\mathrm{mx}}_{4}$
$\Phi_{B32}$	${\mathrm{my}}_{4}-0.1835\;m_{6}-0.1835\;m_{5}-{\mathrm{mz}}_{5}$
$\Phi_{B33}$	${\mathrm{Ia}}_{4}$
$\Phi_{B34}$	${\mathrm{Fc}}_{4}$
$\Phi_{B35}$	${\mathrm{Fv}}_{4}$
$\Phi_{B36}$	${\mathrm{Fo}}_{4}$
$\Phi_{B37}$	${\mathrm{Ixx}}_{5}-{\mathrm{Iyy}}_{5}+{\mathrm{Iyy}}_{6}+0.0520\;m_{6}+0.4560\;{\mathrm{mz}}_{6}$
$\Phi_{B38}$	${\mathrm{Ixy}}_{5}$
$\Phi_{B39}$	${\mathrm{Ixz}}_{5}$
$\Phi_{B40}$	${\mathrm{Iyz}}_{5}$
$\Phi_{B41}$	${\mathrm{Iyy}}_{6}+{\mathrm{Izz}}_{5}+0.0520\;m_{6}+0.4560\;{\mathrm{mz}}_{6}$
$\Phi_{B42}$	${\mathrm{mx}}_{5}$
$\Phi_{B43}$	$0.2280\;m_{6}+{\mathrm{my}}_{5}+{\mathrm{mz}}_{6}$
$\Phi_{B44}$	${\mathrm{Ia}}_{5}$
$\Phi_{B45}$	${\mathrm{Fc}}_{5}$
$\Phi_{B46}$	${\mathrm{Fv}}_{5}$
$\Phi_{B47}$	${\mathrm{Fo}}_{5}$
$\Phi_{B48}$	${\mathrm{Ixx}}_{6}-{\mathrm{Iyy}}_{6}$
$\Phi_{B49}$	${\mathrm{Ixy}}_{6}$
$\Phi_{B50}$	${\mathrm{Ixz}}_{6}$
$\Phi_{B51}$	${\mathrm{Iyz}}_{6}$
$\Phi_{B52}$	${\mathrm{Izz}}_{6}$
$\Phi_{B53}$	${\mathrm{mx}}_{6}$
$\Phi_{B54}$	${\mathrm{my}}_{6}$
$\Phi_{B55}$	${\mathrm{Ia}}_{6}$
$\Phi_{B56}$	${\mathrm{Fc}}_{6}$
$\Phi_{B57}$	${\mathrm{Fv}}_{6}$
$\Phi_{B58}$	${\mathrm{Fo}}_{6}$

### Appendix A.2. Robustness of Identification Methods

The metric relative change is defined as

$$\mathrm{Relative change}=\frac{\Phi_{Bi,c}-\Phi_{Bi,h}}{\Phi_{Bi,c}}\times 100\%$$

where $\Phi_{Bi,c}$ is *i*th identified base parameter in the cold (19∼22 °C) joint temperature condition and $\Phi_{Bi,h}$ is *i*th identified base parameter in the hot (40∼46 °C) joint temperature condition.

**Table A2:** Relative change in the identified parameters in different joint temperature (cold: 19∼22 °C and hot: 40∼46 °C ) condition for conventional and proposed identification method. A background color has been used to signify parameters with relative changes of greater than 20%.

	Conventional:	Conventional:	Conventional:	Proposed:	Proposed:	Proposed:
	Cold	Hot	Relative Change	Cold	Hot	Relative Change
$\Phi_{B1}$	7.8129	8.2334	↑5.3%	7.9853	8.0502	↑0.81%
$\Phi_{B2}$	21.3039	14.6688	↓31.15%	$1\times 10^{-6}$	$1\times 10^{-6}$	→
$\Phi_{B3}$	18.0605	10.8009	↓40.2%	3.0057	2.9737	↓1.06%
$\Phi_{B4}$	0.0068	0.1295	↑18.04%	0.1699	0.1604	↓5.59%
$\Phi_{B5}$	−1.8496	−1.9353	↓4.63%	−1.5784	−1.6053	↓1.7%
$\Phi_{B6}$	0.1143	−0.2168	↓289.68%	−0.3067	−0.2976	↑2.97%
$\Phi_{B7}$	0.0648	−0.8763	↓1452.31%	−0.9783	−0.9511	↑2.78%
$\Phi_{B8}$	0.3234	0.5146	↑59.12%	0.5230	0.5133	↓1.85%
$\Phi_{B9}$	2.1034	5.2002	↑147.23%	5.2119	5.2998	↑1.69%
$\Phi_{B10}$	6.0893	6.1889	↑1.64%	5.9804	6.0565	↑1.27%
$\Phi_{B11}$	0.4989	0.1934	↓61.23%	0.1591	0.1608	↑1.07%
$\Phi_{B12}$	22.8864	18.3729	↓19.72%	1.6388	1.4145	↓13.69%
$\Phi_{B13}$	11.5615	5.5466	↓52.03%	$1\times 10^{-6}$	$1\times 10^{-6}$	→
$\Phi_{B14}$	−0.3248	−1.3259	↓308.2%	−1.0461	−1.1072	↓5.84%
$\Phi_{B15}$	−0.5348	0.1292	↑124.16%	0.7586	0.7770	↑2.43%
$\Phi_{B16}$	−0.0592	−0.0237	↑59.97%	0.0928	0.0932	↑0.43%
$\Phi_{B17}$	0.6998	0.3381	↓51.69%	0.5172	0.5413	↑4.66%
$\Phi_{B18}$	−0.0241	0.1494	↑719.92%	0.0920	0.1234	↓0.81%
$\Phi_{B19}$	1.5420	1.3622	↓11.66%	1.2360	1.1307	↓8.52%
$\Phi_{B20}$	−0.1441	−0.1250	↑13.25%	−0.1469	−0.1363	↑7.22%
$\Phi_{B21}$	−2.2848	−2.3060	↓0.93%	−2.3033	−2.3337	↓1.32%
$\Phi_{B22}$	$1\times 10^{-6}$	$1\times 10^{-6}$	→	$1\times 10^{-6}$	$1\times 10^{-6}$	→
$\Phi_{B23}$	5.3214	4.0017	↓24.8%	1.2387	1.2748	↑2.91%
$\Phi_{B24}$	3.6838	2.2379	↓39.25%	0.2801	0.2256	↓19.46%
$\Phi_{B25}$	−1.8101	−1.2839	↑29.07%	−0.5718	−0.4806	↑15.95%
$\Phi_{B26}$	0.4385	0.2657	↓39.41%	0.3580	0.3685	↑2.93%
$\Phi_{B27}$	−0.0918	−0.0552	↑160.3%	−0.0376	−0.0350	↑6.91%
$\Phi_{B28}$	0.0347	0.0322	↓7.2%	0.0875	0.0889	↑1.6%
$\Phi_{B29}$	−0.0483	−0.0661	↓36.85%	−0.0569	−0.0502	↑11.78%
$\Phi_{B30}$	0.1282	0.1372	↑7.02%	0.2361	0.2097	↓11.18%
$\Phi_{B31}$	0.0398	0.0528	↑32.66%	0.0793	0.0780	↓1.64%
$\Phi_{B32}$	−0.6260	−0.7349	↓17.4%	−0.7718	−0.7869	↓1.96%
$\Phi_{B33}$	0.4611	0.3825	↓17.05%	0.3457	0.3246	↓6.1%
$\Phi_{B34}$	2.6622	1.9719	↓25.93%	0.2740	0.2996	↑9.34%
$\Phi_{B35}$	2.3422	1.3691	↓41.55%	0.6850	0.6392	↓6.69%
$\Phi_{B36}$	0.2623	0.3465	↑32.1%	0.1514	0.1702	↑12.42%
$\Phi_{B37}$	0.1059	0.0997	↓5.85%	0.0331	0.0342	↓3.32%
$\Phi_{B38}$	−0.0113	−0.0096	↑15.04%	0.0019	0.0020	↑5.26%
$\Phi_{B39}$	−0.0132	−0.0099	↑25%	−0.0033	−0.0028	↑15.15%
$\Phi_{B40}$	0.0030	0.0030	→	−0.0075	−0.0086	↓14.67%
$\Phi_{B41}$	0.0995	0.0840	↓15.58%	0.0940	0.0930	↓1.06%
$\Phi_{B42}$	0.0492	0.0356	↓27.64%	0.0293	0.0281	↓4.1%
$\Phi_{B43}$	0.3489	0.3482	↓0.2%	0.3371	0.3488	↑3.47%
$\Phi_{B44}$	0.3579	0.3804	↑6.29%	0.4397	0.4255	↓3.23%
$\Phi_{B45}$	3.2141	2.4732	↓23.05%	$1\times 10^{-6}$	$1\times 10^{-6}$	→
$\Phi_{B46}$	2.2486	1.1070	↓50.77%	0.3842	0.3399	↓11.53%
$\Phi_{B47}$	−0.1617	−0.1197	↑25.97%	−0.0328	−0.0338	↓3.05%
$\Phi_{B48}$	0.0371	0.0222	↓40.16%	0.0050	0.0058	↑16.0%
$\Phi_{B49}$	−0.0029	−0.0011	↑62.07%	0.0070	0.0062	↓11.43%
$\Phi_{B50}$	0.0234	0.0108	↓53.85%	−0.0208	−0.0192	↑7.69%
$\Phi_{B51}$	−0.0116	−0.0067	↑42.24%	−0.0154	−0.0136	↑11.69%
$\Phi_{B52}$	0.1567	0.1045	↓33.31%	0.0705	0.0649	↓7.94%
$\Phi_{B53}$	−0.0147	−0.0066	↑55.1%	−0.0168	−0.0194	↓15.48%
$\Phi_{B54}$	-0.0429	-0.0191	↑55.48%	−0.0371	−0.0357	↑3.77%
$\Phi_{B55}$	0.4289	0.4390	↑2.35%	0.5405	0.5306	↓1.83%
$\Phi_{B56}$	3.2938	2.2460	↓31.81%	0.1426	0.1282	↓10.1%
$\Phi_{B57}$	4.1673	2.3170	↓44.4%	0.6907	0.5733	↓17.0%
$\Phi_{B58}$	0.1594	0.1262	↓20.83%	0.0110	0.0125	↑13.64%
